# Commentary: patient well-being and individual outcomes in the medical practice: impulses from philosophy

**DOI:** 10.1186/s13010-019-0071-x

**Published:** 2019-01-29

**Authors:** Gernot Rüter, Thomas Fröhlich

**Affiliations:** 1 General Practitioner Private Practice, Benningen, Germany; 2 Paediatric Private Practice, Bammental, Germany; 3 Vice President (Western Europe) European Society For Person Centered Healthcare, London, UK

**Keywords:** Existential issues, Doctor-patient relationship, Philosophical concepts

## Abstract

In an everyday private practice setting, regularly also existential topics will emerge from doctor-patient encounters. These are often questions of coping with life and lifestyle. To enable a thorough discussion of such topics, an implicit, and sometimes also explicit reference to a philosophical background is needed. Philosophical concepts to be used in this realm are discussed. An individual patient-doctor interaction is used as an example to demonstrate the doctor’s choice of hermeneutical and phenomenological philosophical concepts.

## Background

To ensure a principal level of quality in healthcare, standardization, evaluation and a continuous re-evaluation are inevitable prerequisites. The limits of this necessarily schematizing and generalizing approach become evident as soon as also existential topics need to be discussed. Sudden onset of disease, or a near death may cause the patient and the doctor to go into individual details not covered by general schemata as provided by evidence based medicine. The situationally-adapted interactions need instead a root in concepts concerning meaning of life, including, if applicable, also non-religious and religious beliefs, experiences and convictions. To this aim, and in our opinion, reference also to philosophy is inevitable. Here, it is preferably hermeneutical, phenomenological and existential philosophy to be referred to. As is obvious, such concepts cannot be applied in a schematic way. Instead, the logical level to be addressed differs from patient to patient and moment to moment. Healthcare providers should become familiar with this form of not schematized reasoning going beyond application of therapy guidelines.

### The existential level

In a typical German general practitioner’s private practice one will have some 40–80 doctor-patient encounters per day, adding up to some 1500 patients treated within a three-month period. This holds true for the first author’s practice, and – with lower numbers - also to the second author’s paediatric practice. The doctor knows many life histories of these persons, as well as social aspects of their lives. From time to time, patients show up with a development of a disease that causes a change on the existential level. This individual development is also referred to in form of individual “cases”, but with a deeper rooting in the patient’s existence. In the present article, it will be shown how a doctor gets involved in these processes happening on an existential level. The background of the approach presented is both individual from the doctor’s side and philosophical [1–6]. A search for philosophical concepts addressing doctor-patient relationships did not produce simple to use concepts. Instead, a variety of philosophical concepts must be addressed to investigate the different components of an encounter on a not only technical, but also existential level.

Here, the “cultural” problem in medical education/training is its almost exclusive orientation on objectified organs, diseases, and disease-focused surgical or medical treatment, and not on the “person” suffering from these diseases. The wider personal, individual horizon comes into sight only in moments of existential crisis. With the medical training described here, doctors only rarely have professional training to cope with such challenging situations. One form of training is the participation in Balint groups, a well-established form of moderated discussions of healthcare workers, also offering elements of self-experience.

We now give an example of the emergence of existential topics within a normal course of a workday. We use the form of a “case history”, stating that a general practitioner’s work is focused on persons, individual moments, life histories and corresponding narratives, summarised as “cases”, and not only on diseases.

### Convergence of burdens

A paediatric nurse, in the years before only asking for therapy because of pain in the back, seems to be determined to discuss further problems. She explains that she has lost her job, and at the same time will have to move home, because the landlord wants to modernise it. At the same time, her stepfather, whom she calls “Father” has become sick with a life-threatening disease, and is expected to die soon. Her relationship with this stepfather is much stronger than the relation to her mother had been or to her real father whom she had never really experienced as a father as he was not interested in establishing a relationship to his daughter at all. The mother died ten years ago.

As if this would not be challenging enough, also her partner told her he would end their relationship. In kind of a self-alienation and in a frightened state, she observes changes in her behaviour now. She has started to drink wine, up to one bottle per day, and also alcohol in form of spirits. Even more irritating and scaring is what she describes as impulsive high-speed biking at street crossings, even at a risk. In mentioning these frightening observations, the patient also tells the doctor she has already planned her own funeral precisely.

The doctor allows the dialogue to develop, and offers further encounters, if needed. Within a short period, there are four further encounters, each lasting some 20 min. Within this initial sequence, the doctor assures the patient that he would deeply regret the patient’s death, and asks if there is anything on the horizon to give her hope. The patient doesn’t see any chance for hope, but tells the doctor that also others let her know that they would be very sad if she died. At this moment, the tension, as experienced and interpreted from the doctor’s side gets less intense, allowing for a more rational discussion concerning abuse of alcohol, and the patient’s implicit suicidal behaviour.

During the course of interventions, external help is accepted, first in form of a stay in a hospital offering therapy of psychosomatic diseases, then in form of treatment in this hospital as a daytime or outpatient. Later, the patient had a stay in a further hospital, offering rehabilitation. It took two years to stabilise the psychic situation of the patient including addressing her problems at work, and housing referral.

### Philosophical concepts to apply

In a search for philosophical concepts possibly relevant in understanding this long lasting and complicated, multi-facetted interaction form from the doctor’s and the patient’s side, at first existential philosophy might be taken into account. With its origins in the work of philosophers like Søren Kierkegaard whose philosophy has been characterised by the German philosopher Gerhard Gamms as conceptualising the fact of an individual’s existence as experienced from the individual’s point of view. Gamms states that this is not an abstractive approach, but a detailed, specific, tangible topic [7]. Enid and Michael Balint, as Hungarian-British psychoanalysts aimed to combine this focusing and the abstractive nosological approach in coining the term “overall diagnosis”. It is a precise diagnosis, with the precision not limited to physical, but extended to “psychological and social components of the patient’s presentation” (cited from [8]).

One of the members of the first Balint group, M. B. Clyne, who the first author G. R had the chance to meet in person wrote, that the overall diagnosis is meant to give an overview concerning the bodily and emotional situation, and the relation to oneself and others, including the doctor [9].

In enacting such an overall diagnosis, several steps have to be taken before or within continued interaction. First, the doctor must be allowed and dare to be involved. This involvement creates not an only detached, observational position, but at the same time exerts a professional control and self-awareness of being engaged. This is needed to not tacitly identify with the patient’s point of view not adding anything additional and not transcending the patient’s momentary position and view.

The German Neo-Phenomenologist Hermann Schmitz has investigated the existential, hence also bodily aspects of getting involved. It is not a matter of representing an other’s feelings in oneself, but instead a mutually embodying being-contained and being jointly immersed in a non-spatial atmosphere with no finite limits, as Schmitz tells [10]. He discerns two forms of “Einleibung” (embodiment); the solidary and the antagonistic one. The latter explicitly addresses another person aiming to make them also immerse themselves in the uttered and communicated feelings. This happened in the case of the patient mentioned above; she got immersed in the non-bodily, friendly-supportive embrace of the doctor’s saying he would regret if she would die.

### Hermeneutics

A prerequisite to professionally controlling this getting involved is applying an explicit hermeneutic, such as presented by the German philosopher Hans-Georg Gadamer [11]. He distinguishes four “moments” or components of hermeneutical understanding; a cognitive, a practical (knowing and understanding how to do something), an anticipative (expecting and pre-empting a football player’s run), and a communicative mutual understanding. The latter corresponds to the being-jointly-immersed in a shared atmosphere, as described by Hermann Schmitz. This may be achieved stepwise, but also in a sudden moment, spontaneously emerging from tacit, hidden roots, like a flash, and Michail Balint hence suggested a technique, supporting emergence of such communicating moments [12].

This hermeneutic approach conceptualises a person as both being exposed to, and actively realising one’s own existence. Whereas the German philosopher Martin Heidegger puts more stress on the “Geworfen-Sein” (being thrown into the world), the French philosopher Jean-Paul Sartre also put an accent on the active moment of self-determination, concerning one’s position in the world. Heidegger would not stress the directing potential of men that much, but prefers to see them as a shepherd of their own existence. This is a more humble approach, nearer to everyday experience of an empirical “muddling through”, without obvious paths simply needing to be chosen.

### Doctor’s life gets involved, also

Concerning a principal, not only cognitive involvement of a doctor in her or his patient’s life, it is not only the patient’s life being addressed in an encounter, but also the doctor’s life. If such an involvement is accepted from the doctor’s side, it will become an integral part of her or his work. Work and life, as seen from the doctor’s side then are not completely separated entities; instead, they differ only in form of accents. The modern ideal of a work-life balance, as aimed for also in medical education denies, or too less accentuates the principal, not only cognitive involvement of a doctor in her or his patient’s life.

As obvious from the numbers of contacts per day, a deep involvement from a doctor’s side will not be the default status, but the exception. Yet, it inevitably forms a part of the “profession”, seen as an existential commitment to the “*professio*”, similar to that of firemen or policemen ready to give their lives, if needed. And, in fact, it is this simultaneously spontaneous and professionally controlled as well as adapted involvement, which makes work in healthcare different from many other jobs.

### How to address the existential level in a healthcare setting

In the “case” described above doctor’s psychic involvement is not dealt with explicitly. Instead, it may be inferred from his notion that he would regret it if the patient died, indicating a feeling that would be caused in him in the case of loss of his patient. What the doctor expresses here is not his specific role, but relates instead to him as a human being too. On this existential level, there is a basic human equality underpinning the distinct roles played by the interacting persons. To switch from the role-limited professional to the main human level is usually a spontaneous act. As discussed before, to change the level in a not only spontaneous, but a professionally adapted form is a change, which may be supported by reference to philosophical concepts. Common reasoning of emotional, and also existential topics in Balint groups gives additional support in addressing professionally the existential level, if needed.

If patient’s life, as expressed by her or himself elicits a corresponding feeling and reasoning in the doctor, it then may be addressed frankly in an explicit, appropriate, adapted, at least partially deliberate and controlled way. This will automatically change the encounter from one of actors caught in their roles into one of two human beings, exposed to the same existential topics in principle, be it to a different extent in the moment. As soon as typical human exposedness is concerned, it is an eye-to-eye contact, and the view is not deviated by focussing on sheets of paper, or computer screens, instead.

Before being a patient, the individual concerned has been a person, just as the doctor returns to being when leaving the private practice or the hospital. To dive into the depth of the existential level is hence a return to the states before and after. It may have been a vague or a specific insecurity, which caused the person to adopt the role of a patient. It is seen as the doctor’s role’s duty to re-install certainty, be it with an alleviating, or a clearly identifying statement. During the role-to-role encounter, the basic task consists in generation of security.

To leave the shelter of the role in favour of a somehow unpredictable, course of life and existence, installs a main threat, which is usually avoided for emotional reasons. In a tacit agreement in a secret plot, not only the doctor, but also the patient may hence prefer not to touch the level of existential topics. Both may agree on focusing on numbers, curves, images and other strictly objective features, in form of present evidence in mostly statistical figures. Sometimes, this may have the character of “whistling in the dark”, and a role-play meant to avoid the actual existential level in the need to show concern. The doctor and the patient may be caught in collusion, to use a term introduced by the psychotherapist Jörg Willi [13].

### The figs

**Fig. 1:**
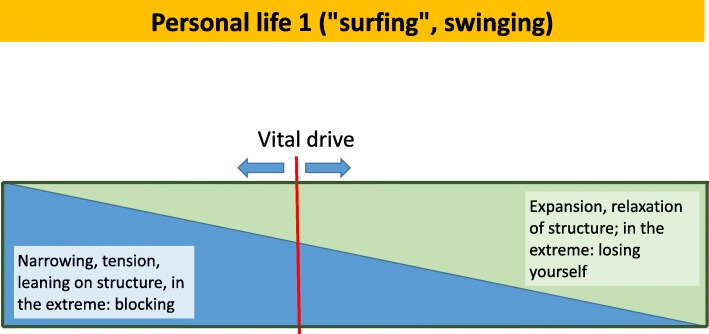
Personal life 1 (“surfing”, swinging)

A not too difficult-to-pass entrance into the realm of existence and the corresponding human eagerness to create good and successful lives is the addressing of simple and spontaneously experienced, momentary bodily feelings, such as being compressed, or alleviated. The previously mentioned phenomenologist Schmitz has dealt with these feelings in distinguishing two principal “directions”; a narrowing inward and an expanding, alleviating outward one. Since this, to a greater or lesser extent, refers to everyone’s everyday experience, it is easy to jointly focus on the topic of tension versus expansion, and this way to automatically address the level of existence and existential feelings. The two opposite directions are depicted in Fig. 1, as transiently chosen directions of a vital drive, as one may call it.

**Fig. 2:**
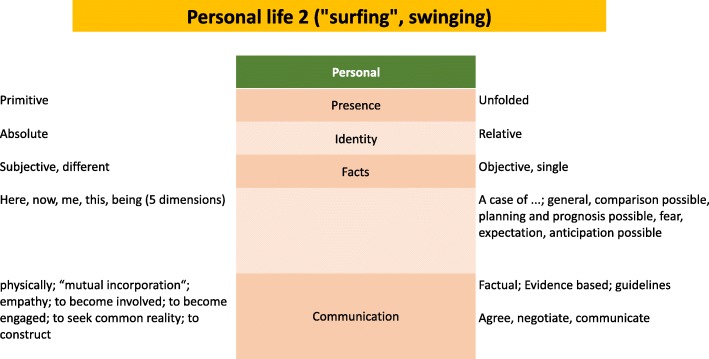
Personal life 2 (“surfing”, swinging

Figure 2 introduces some more elements of the two distinct paths. These may be used alternatively, as if one would continuously perform a swinging movement. The inward move leads to regression, the outward to an unfolding, allowing for a detachment from the momentary situation. As is obvious, the role-centred setting goes into this detached mode, and hence prefers objective, single facts, interconnected in a logical, causal network. But also the opposite direction has to be taken into account, as outlined before, since this goes down to the roots and an existential exposedness that cannot be deliberately or freely be changed. The middle column marks potential starting points that allows one to go in both directions alternatively, and hence opens the horizon to contain either the rooted or the uprooted, objectified position.

The figure also summarises what can be learned from the phenomenologist Schmitz’ concept of what constitutes a person in a not digitalising approach. Here, each component is seen as sensitive, interacting adaptively, continuing to be different in an also continuous adaptation to individual other such constituents, and to the set of such, as composed momentarily.

**Fig. 3:**
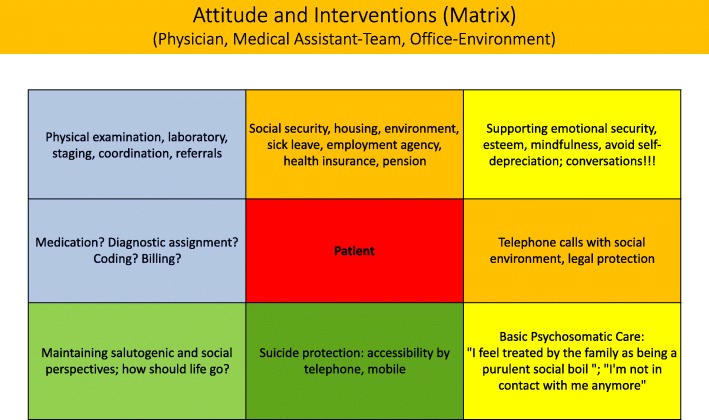
Attitude and interventions

Figure 3 puts the patient in the middle. It resumes the spectre of potential securing approaches typical, but not exclusive for a general practitioner in private practice. Combined, they create a protective shield with also existential topics and processes taken into account, such as bringing an end to one’s life, and psychosomatic issues.

**Fig. 4:**
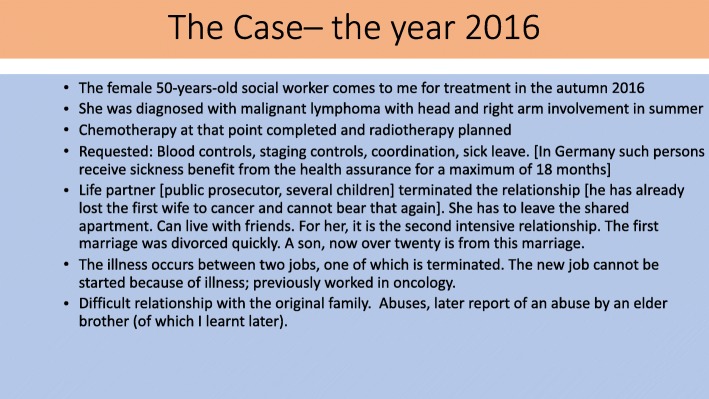
The Case – the year 2016

**Fig. 5:**
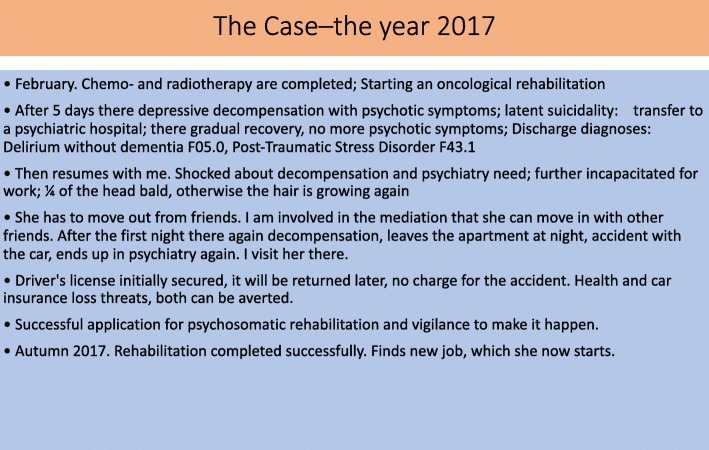
The Case – the year 2017

Figures 4 and 5 summarize a further “case” to show the different interventional paths of Fig. 3 used to co-create an alleviating transient solution.

**Fig. 6:**
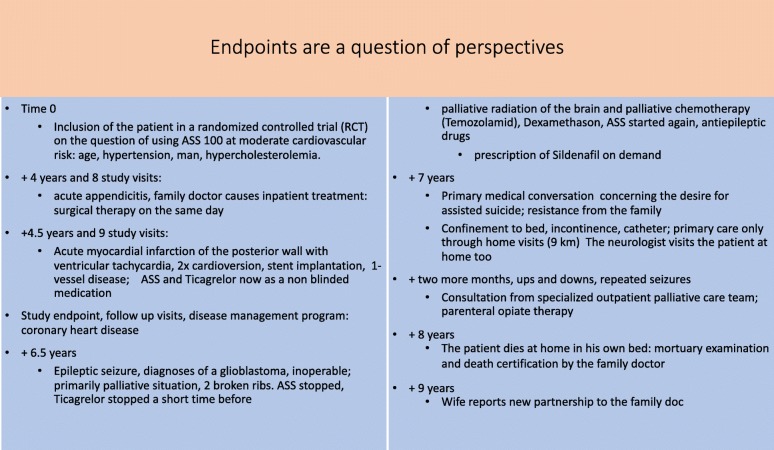
Endpoints are a question of perspectives

Figure 6 shows what happens behind the curtain of a randomised controlled trial structure; it is real life, and not only a small window opened to collect statistical data. As well, it shows that the doctor, exposed to the patient’s course of life is immersed in a much deeper and broader way than presented in a selective reference to a decontextualised study object / participant only. It also helps the understanding that the results of such objectifying procedures can only form a part of the doctor’s interventional repertoire. Especially, existential processes such as dying are depicted in the detached form of numbers, such as number of persons having died in course of the study.

**Fig. 7:**
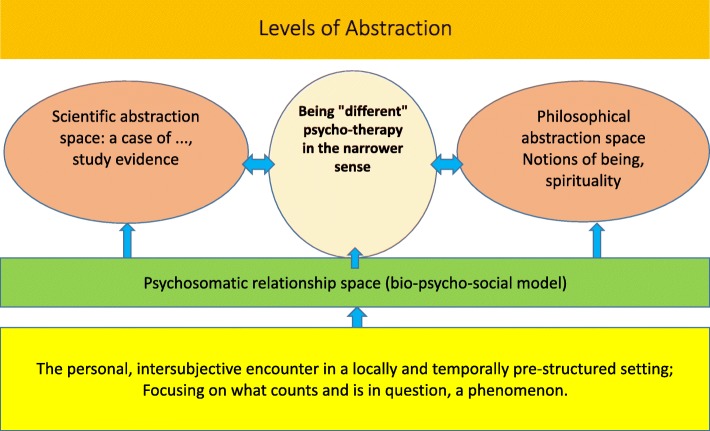
Levels of Abstraction

The last Fig. 7 may be read in different ways. One may start with the momentary focus, an individual patient’s “being different and specific” for the time being, and move from that focus into its realms, depicted as semantic abstraction spaces or levels. Or, one may follow an inductive path that starts with the present situation of both participants of the doctor-patient encounter, and its details and specifics. What is clear, in every instance, is the multi-level or polycentric character of any patient-doctor interaction [6]. Exclusive focusing on only one level, and selective moving in only one abstraction space, such as the scientific does not cover the factual spectre of spaces and levels inevitably active in the encounter.

## Conclusions

If a patient’s existential issues form a relevant part of the doctor-patient encounter, the doctor may either ignore this level, or allow himself/herself to get involved. Professional control of involvement, as well as a not-ignorant approach will be ensured by additional reference to philosophical concepts. The German philosopher Hermann Schmitz has discussed the opposing poles of threatening narrowing and relief by expansion from a phenomenological point of view. The corresponding concept of a “Leib” as different from a body in conventional terms has been also been discussed by the American philosopher Richard Shusterman, who suggests the use of the term “soma” instead of body [14].
